# Mapping the colorectal tumor microbiota

**DOI:** 10.1080/19490976.2021.1920657

**Published:** 2021-05-25

**Authors:** CL Murphy, M Barrett, P Pellanda, S Killeen, M McCourt, E Andrews, M O’ Riordain, F Shanahan, Pw O’Toole

**Affiliations:** a APC Microbiome Ireland, University College Cork, National University of Ireland, Cork, Ireland; b Departments of Gastroenterology and Medicine, Cork University Hospital, Cork, Ireland; c School of Microbiology, University College Cork, National University of Ireland Cork, Ireland; d Department of Colorectal Surgery, Mercy University Hospital, Cork, Ireland; e Department of Colorectal Surgery, Cork University Hospital, Cork, Ireland

**Keywords:** Colorectal, cancer, microbiome, tumor, gut

## Abstract

The gut microbiome in patients with colorectal cancer (CRC) is different than that of healthy controls. Previous studies have profiled the CRC tumor microbiome using a single biopsy. However, since the morphology and cellular subtype vary significantly within an individual tumor, the possibility of sampling error arises for the microbiome within an individual tumor. To test this hypothesis, seven biopsies were taken from representative areas on and off the tumor in five patients with CRC. The microbiome composition was strikingly similar across all samples from an individual. The variation in microbiome alpha-diversity was significantly greater between individuals’ samples then within individuals. This is the first study, to our knowledge, that shows that the microbiome of an individual tumor is spatially homogeneous. Our finding strengthens the assumption that a single biopsy is representative of the entire tumor, and that microbiota changes are not limited to a specific area of the neoplasm.

## Introduction

Colorectal cancer (CRC) is the second largest cause of cancer death in the United States^1^. Sporadic CRC arises after a series of cumulative genetic mutations,^2^ with a 10-year progression from adenoma to CRC.^3^ The microbiome is distinctly different in biopsies of CRC and adenomatous polyps,^4,5^ leading to an updated hypothesis that microbial changes^6^ and secondary consequences for immunological cell signaling^7^ may play a role in tumor progression. Bacteria are an established risk factor for cancer, such as *H. pylori*-related MALT lymphoma and gastric carcinoma.^8,9^ In particular, several individual microbes such as *Fusobacterium nucleatum*^10^ *and Escherichia coli*^11^ have been implicated in the pathogenesis of colorectal cancer, but a cause–effect relationship has not been established; rather, microbes and their metabolomes represent complex collections of gene networks that interact bidirectionally with cancer cells.^12^

CRC-associated microbiota is characterized by a reduced alpha diversity compared with healthy controls.^13^ Patients with CRC^4,14^ or adenomatous polyps^4,15^ show also distinct qualitative differences in both the microbiome and metabolome in fecal^16,17^ and biopsy samples^4,14^ compared with healthy controls. In these studies, the microbiota associated with cancerous and non-cancerous tissues within the same individual did not differ significantly^4,14^ which suggests that in CRC, a global microbial ecosystem change occurs throughout the colon.^4,18^ However, the microbial alterations differ between proximal and distal cancers.^4^ These compositional changes often represent a relative over-abundance of oral bacteria, which are hypothesized to organize into biofilm-like structures^19^ on the tumor and on the right side of the colon.^4,20^ We have previously described that CRC patients can be stratified into four groups based on bacterial co-abundance groups (CAGs) that link distinct mucosal gene-expression profiles^4^ with similar networks of oral-based bacteria found in the gut mucosa and oral mucosa.^18,20,21^

Distinct morphological and phenotypical differences exist within and between colorectal tumors.^22^ Classification systems such as NICE,^23^ Paris^24^ and Kudo^25^ use macroscopically visible differences in lesions to stratify malignant potential^24^ or stage neoplastic tumors^26^ detected at the time of endoscopy. Similarly, the World Health Organization (WHO) has classified the appearances of colorectal tumors at surgery into four groups: exophytic, endophytic, diffusely infiltrative and annular, with the recognition that significant overlap occurs between these categories.^27^ Macroscopic phenotypes may also be an overall predictor of genetic alterations and DNA methylation in a colorectal tumor.^28^ Intra-tumoral heterogeneity for both genetic and epigenetic factors in CRC are also evident.^29^

Untargeted colonoscopy biopsies or untargeted segments of resected tumors have been used in most studies of CRC microbiota.^4,14,30,31^ Given the histologic and genetic intratumoral heterogeneity^32^ of CRC, topographic variance in the microbiota of a single tumor may be a confounding factor. Therefore, we undertook the first study that aims to investigate the intratumoral microbial heterogeneity and its comparison with adjacent proximal and distal non-cancerous tissue.

## Results

Five patients were recruited to the study, four males and one female, with a mean age of 72 ± 6.7 years with demographics shown in Table 1. All patients had a diagnosis of colonic adenocarcinoma within the previous 1–2 months. Seven samples were obtained from each individual comprising normal tissue proximal to the tumor (biopsy 6), normal tissue distal to the tumor (biopsy 5), a central tumoral biopsy (biopsy 5) and four peripheral tumor biopsies (biopsies 1–4). The tissue microbiome was profiled by 16S rRNA gene amplicon sequencing.

**Table 1.** Patient characteristics

Patient	GT 001	GT 007	GT 009	GT 010	GT 011
**Type of neoplasm**	Adenocarcinoma	Adenocarcinoma	Adenocarcinoma	Adenocarcinoma	Adenocarcinoma
**Tumor location**	rectum	transverse colon	sigmoid colon	caecum	ascending colon
**Stage of neoplasm**	T3N0M0	T3N0M0	T3N1M0	T3N1M0	T3N0M0
**Time since diagnosis (months)**	1	1	1	1	2
**Type of surgery**	Anterior resection	Right hemi-colectomy	Anterior resection	Right hemi-colectomy	Right hemi-colectomy
**Bowel Prep**	Moviprep	Moviprep	Moviprep	Moviprep	Moviprep
**Alcohol intake per weeks**	10 units	none	3 units	none	none
**Smoking status**	Current (2/day)	Ex-smoker (10/day x20years)	Ex-smoker (20/day x40years)	Non smoker	Ex-Smoker (10/day x35 years)
**Probiotic use**	No	No	No	No	No
**Antibiotic exposure**	No	Yes	Yes	Yes	Yes
**Antibiotic regime used at surgery**	N/A	IV co-amoxiclav and metronidazole	Oral metonidazole and neomycin	Oral metonidazole and neomycin	IV co-amoxiclav and metronidazole
**Diverticulae**	no	no	no	no	no
**Medical comorbidites**	none	Hypertension, NIDDM	NIDDM, obstructive uropathy	Hypertension, anemia	Epilepsy, NIDDM, hypertension, hyperlipidemia
**Medications**	nil	aspirin, ramipril, esomprazole, atorvastatin, empagliflozin, metformin	atorvastatin	ramipril, lercanidipine, ferrous fumerate	bisoprolol, ezetimibe, rosuvastatin, hyoscine butylbromide, esomprazole, lercanidipine, carbamazepinesitagliptin, metformin

Footnote: n = 4 males, 1 female, with a mean age of 72 ± 6.7 years

**Figure 1.**
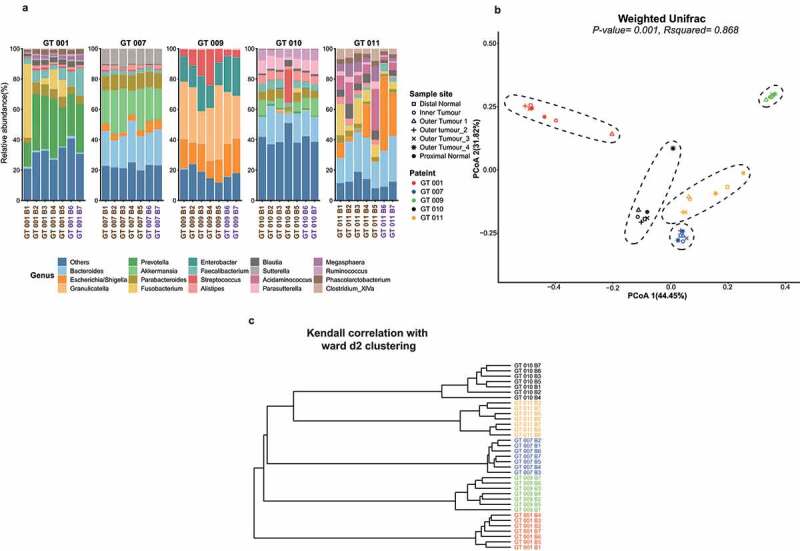
Microbiome relatedness of biopsies within Individuals. (a) Taxonomic bar plot of the proportional relative abundance of genera. “Others” is a grouping of genera with less than 1% abundancy across the samples as well as unclassified genera (b) PCoA plot representing weighted Unifrac distances. Biopsy location is represented by shapes while colors represent individual patients. Utilizing the R package ggforce v0.3.1, ellipses were estimated using the Khachiyan algorithm. R-squared (R^2^) and *p*-values were calculated using Permutational Multivariate Analysis of Variance (PERMANOVA) via the R package vegan v2.4–2. (c) Dendrogram representing Kendall correlation with ward d2 clustering. Samples are colored by individual

The microbiome composition was highly similar among samples within a particular individual (Figure 1a). The genus level composition differed significantly between patients (Figure 1a) but was remarkably similar within a single subject, both on (biopsy 1–5) and off the tumor site (biopsy 6 and 7). This was reflected in beta diversity distance metrics wherein samples are clustered by individual rather than biopsy site as represented in Principal Co-ordinate Analysis (PCoA) plots (Figure 1b). The identity of the patient from whom the biopsy was taken was associated with the top four PCoA axes which collectively explained >90% of variance (see Supplementary figure 1S, Supplementary table 1). However, there was no association between any of the top 10 PCoA axes, which collectively explained ~99% of the variance, and sample site (Supplementary table 2). We employed permutational multivariate analysis of variance (PERMANOVA) to calculate the association between sample metadata factors and the global microbiome structure as defined by the beta-diversity distance matrixes. A strong association between the biopsy patient origin and the microbiome was identified (Figure 1b, Supplementary table 3). However, we did not detect any statistically significant association between the global microbiome structure and the sample site (Supplementary table 4). We next performed a patient-specific rank sum normalization on all samples to reduce the impact of patient bias. We performed a PERMANOVA on this transformed data to test for a significant association between location and the beta diversity metrics. However, we did not find a significant association (Supplementary table 5).

The beta diversity clustering data were supported by hierarchical clustering in which the topology of the dendrogram was clearly dictated by the subject identity rather than biopsy site (Figure 1c). Within subjects, there was no reproducible pattern of microbiota relatedness with anatomical origin that was replicated across subjects (Figure 1c).

**Figure 2.**
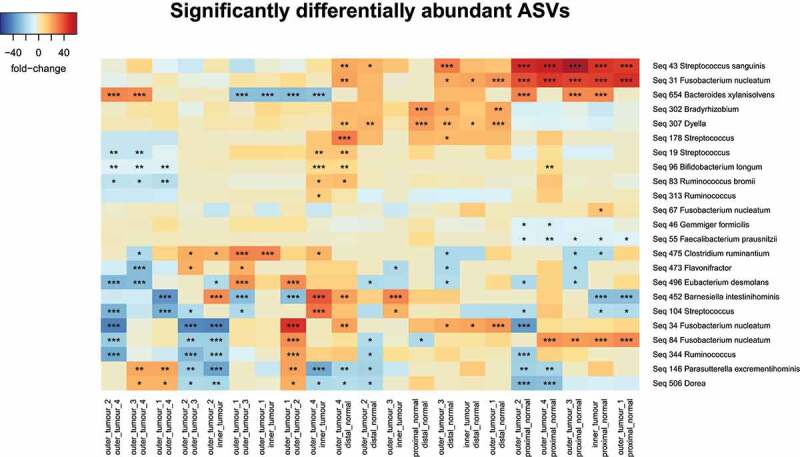
Differentially abundant ASVs. Heat plot displaying differentially ASVs between each pairwise comparison of every sample sit. Column names indicate which pairwise comparison. Row names display ASVs with which taxa it was assigned too. Only ASVs which could be assigned to the Genus level displayed. Stars indicate *P*-value. *<0.05, **<0.01 and ***<0.001

Samples were pooled based on biopsy site and pairwise analysis was performed for each sample pair within the biopsy site. Differential ASV abundance was not detected with respect to anatomical site when we applied paired sample Wilcoxon test with Benjamini-Hochberg adjustment for multiple comparisons (Supplementary table 6). We next utilized DESeq2 which has been demonstrated to be sensitive when applied to small sample sizes.^33,34^ We identified a number of differentially abundant ASVs between sample-sites while controlling for which patient the biopsy originated from (Figure 2). Notably, a number of ASVs assigned to the oral species *Fusobacterium nucleatum*, were observed to be enriched on tumor samples relative to undiseased disease (distal normal and proximal normal). In particular, Seq 31 was identified to be enriched in 5/5 proximal tumor biopsies relative to a healthy distal biopsy and 4/5 tumor biopsies relative to the healthy distal biopsy.

**Figure 3.**
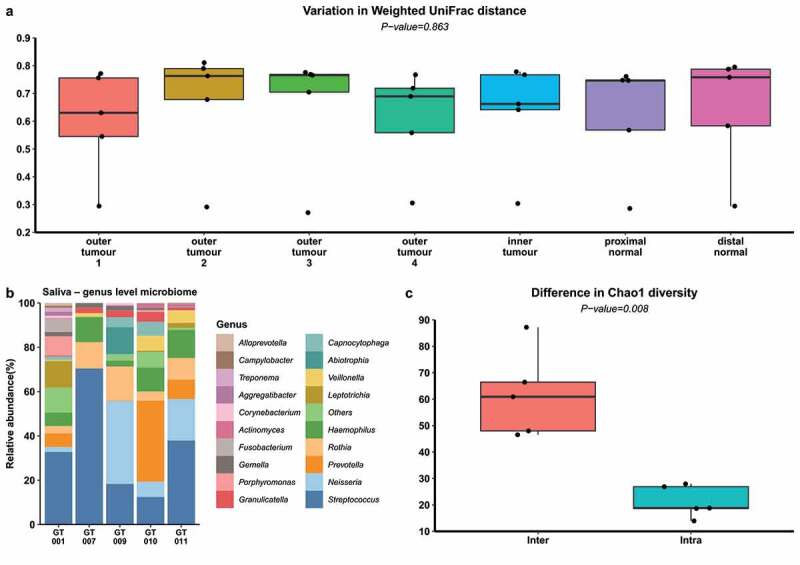
(a)Bar plot of the difference in Beta-diversity distance between the microbiota of indicated biopsy sites and paired buccal swab microbiota from the same subject. Kruskal–Wallis test was used to calculate *p*-values. (b) Taxonomic bar plot of the proportional relative abundance of genera of oral samples. “Others” is a grouping of genera with less than 0.25% abundancy across the samples as well as unclassified genera. (c) Bar plot displaying the difference between Inter-individuals versus Intra-individual variation in alpha-diversity (Chao1)

Previous studies have indicated that oral microbes can translocate from the oral cavity to the gut.^35^ Furthermore, CRC tumor microbiota is enriched with oral taxa.^20^ For these reasons, the buccal swab microbiota composition was analyzed and compared to that of the respective subjects’ biopsy sites as a function of beta diversity distance (Figure 3a, 3b, Supplementary Figure 2S). This analysis revealed that the microbiota of all the biopsies were equally distant from the oral microbiota in all the subjects.

The sequencing depth of the samples allowed for a thorough investigation of alpha diversity, that is microbial richness and evenness (Supplementary table 6, Supplementary Figure 3S). Considering all biopsies from each sample sites examined, the difference in alpha diversity of the biopsy microbiota datasets as measured by five different indices was significantly greater between any two individuals than it was within individuals (Figure 3c, Supplementary figure 4S).

## Discussion

Many studies have profiled the microbiome in CRC using cancer tissue^4,14,30,31^ from a single biopsy assuming that the microbiome profiled in this single specimen was representative of the tumor as a whole. This study confirms that this is a valid assumption.

Given the macroscopic and microscopic heterogeneity of CRC tumors, it may seem surprising that the microbiome of an individual tumor is very similar throughout the entire tumor tissue, as shown in this study. In contrast, significant differences were noted in the genus level abundance of particular taxa in the microbiota sequenced from biopsy samples from five individuals in the study. These variations are probably due to the differences of tumor location (Figure 1) as has been previously reported,^4,30^ as well as to other factors such as antibiotic exposure^36^ and diet,^37^ which are known to alter the baseline microbiome.

Interestingly, as we showed in a previous study,^4^ paired samples of un-diseased tissue proximal and distal to the tumor harbored the same microbiota with respect to dominant taxa and their relative abundance. Previous work has demonstrated the presence of anaerobic oral bacteria on the colorectal tumor mucosa^20,31^ consistent with the notion of a biofilm of pathologic bacteria forming^38^ and seeding on the tumor. In the current study, various distance metrics did not show that any particular site was closer to the oral microbiome. However, we did detect specific oral-associated taxa such as *Fusobacterium nucleatum* and *Streptococcus sanguinis* overrepresented on tumor sample sites. Indeed, from the growing catalog of microbes associated with CRC many of these microbes belong to oral-associated taxa including *Fusobacterium, Porphyromonas, Gemella, Streptococcus* and *Leptotrichia*.^39^ Two routes of translocation of oral microbes to the colon have been proposed: 1) though the gastrointestinal tract and 2) through circulatory system.^35,40^ Both *Fusobacterium nucleatum* and *Streptococcus sanguinis* have been observed to cause endocarditis, demonstrating the potential to travel through the circulatory system.^41,42^ *Fusobacterium nucleatum* is of particular note due to the growing body of evidence of its mechanistic role in the oncogenesis of CRC.^41^

There are some limitations to this study. The sample size of five patients is small, but tumor tissue within each individual was extensively biopsied to capture macroscopically morphologically different areas such as ulcerated and non-ulcerated tissue. Four individuals were treated with antibiotics prior to or during the procedure as per hospital protocol. Similarly, all patients had bowel preparation on the day prior to their surgery which is known to alter the microbiome.^43^ However, in this study, each individual was taken as a separate entity, therefore acting as an internal control and comparator and it is assumed that these modifiers of the microbiome affected the microbiome as a whole.

The global burden of CRC is increasing and this disease is a significant contributor to cancer deaths^1^. Prospective trials are ongoing that incorporate microbiota analysis with other factors as part of the investigative assessment and staging of cancer^44^ and to predict CRC outcomes.^45^ Through demonstration of microbial homogeneity within an individual tumor and in the adjacent normal tissue, this study helps validate the methodology of sampling tissue going forward for these and other indications.

## Patients and Methods/Materials and Methods

### Patient recruitment

A total of five patients who were scheduled for colonic resection for colorectal cancer as part of their standard of care at Cork University Hospital and Mercy University Hospital, Cork were recruited for the study. Patients were labeled as GT (Geography of Tumor) 001,007,009,010 and 011. Recruitment to the study took place from February 2019 to June 2019. Ethical approval was granted by the Clinical Research Ethics Committee of the Cork Teaching Hospitals (Cork, Ireland). The study was conducted in accordance with the ethical principles set forth in the current version of the Declaration of Helsinki, the International Conference on Harmonization E6 Good Clinical Practice (ICH-GCP). Exclusion criteria included a history of inflammatory bowel disease or irritable bowel syndrome, a significant acute or chronic coexisting illness and neoadjuvant chemotherapy or radiotherapy. All patients received a macrogol preparation preoperatively. A single dose of oral metronidazole and neomycin were administered to two patients preoperatively and two other patients received intraoperative intravenous co-amoxiclav and metronidazole as per hospital protocol. The fifth patient took no antibiotics. None of the patients had probiotic exposure preoperatively.

A mouth swab was taken from patients in the preoperative room prior to anesthesia and snap frozen. Immediately after removal from the patient, the ex-vivo specimen was anatomically orientated, was dissected and the tumor was exposed. A representative tissue biopsy from each of the four quadrants of the tumor was taken in a clockwise manner starting at 12 o’clock. Tissues from the central area of the tumor plus two biopsies of adjacent macroscopically normal tissue 10 cm proximal and distal to the tumor were taken. A different set of sterile instruments was used for every biopsy taken and for each individual. This ensured there was no transfer of bacterial material from sample to sample within or between individuals. Samples were snap frozen in cryotubes and transferred immediately for storage at −80 °C.

### DNA extraction and 16S RNA amplicon sequencing

Genomic DNA from biopsies was extracted using the AllPrep DNA kit from Qiagen. When preparing each sample, approximately 20 mg in total of tissue was dissected in small fragments from around the biopsy and pooled. These pooled fragments were then added to a bead beating tube containing sterile beads and 600 µl of buffer RLT plus was added. Samples were then homogenized for two 15 sec at full speed pulses in a MagnaLyzer (Roche, Penzberg, Germany) with rests on ice between pulses. The rest of the DNA extraction was carried out according to the Qiagen AllPrep DNA/RNA extraction kit. Oral genomic DNA was extracted using Qiagen DNeasy PowerSoil Kit following the manufacturer’s instruction.

### Library preparation and sequencing

The 16S rRNA gene was amplified using primers for the V3-V4 region; forward, TCGTCGGCAGCGTCAGATGTGTATAAGAGACAGCCTACGGGNGGCWGCAG-3′ and reverse, 5′-GTCTCGTGGGCTCGGAGATGTGTATAAGAGACAGGACTACHVGGGTATCTAATCC-3ʹ. DNA was normalized to a concentration of 10 ng/µl and 10 µl DNA was added per 30 µl PCR reaction. The PCR thermocycler protocol was as follows: Initiation step of 98°C for 3 min followed by 30 cycles of 98°C for 30 s, 55°C for 60 s, and 72°C for 20 s, and a final extension step of 72°C for 5 min. Indexes were subsequently added to the purified amplicons according to Illumina 16S Metagenomic Sequencing Protocol (Illumina, CA, USA). Libraries DNA concentration was quantified using a Qubit fluorometer (Invitrogen) using the ‘High Sensitivity’ assay and samples were pooled at a standardized concentration (80 ng of each sample). The pooled library was sequenced at Eurofins Genomics/GATC Biotech (Konstanz, Germany) on the Illumina MiSeq platform using 2 × 300 bp chemistry. All the samples in this study were prepared in the same library and sequenced together.

### Bioinformatics analyses

Raw data was imported into R v3.5.3 for processing and analysis. Paired reads were quality filtered, trimmed, merged and Amplicon Sequence Variants (ASV) inferred using the R package dada2 v1.12.1. The following parameters were used for the filterAndTrim function; filtRs,trimLeft = c(19,21),maxEE = c(2,2), truncLen = c(260,230). Taxonomic classification was performed using the RDP naive Bayesian Classifier within the dada2 against the Silva v132 database. Alpha diversity was calculated from the ASV table using QIIME v1.9.1 as previously described in Kuczynski et al.^46^ Samples were rarefied to 7000 reads in order to calculate alpha-diversity. QIIME v1.9.1 and the R package vegan v2.5.6 were used to infer β-diversity metrics.^47^ β-diversity was visualized via principal coordinates analysis (PCoA) plots whose coordinates were identified using the Ape package v5.1. The adonis() function within the R package vegan (v2.4–2) was used to perform permutational multivariate analysis of variance (PERMANOVA) difference in paired biopsy-buccal distance was assessed using paired Wilcoxon test. DESeq2 (v1.28.1) was used to identify differentially abundant taxa from the microbiota dataset.^33^ Differences between inter- and intra-alpha diversity were tested using Wilcoxon signed-rank test.

### Contamination control

We first carried out mock extractions to detect reagent-associated contamination from the two kits used in this study (Supplementary figure 5S). Further, we also carried out PCR controls, i.e., water, to detect contamination specific to the polymerase (Supplementary figure 5S). These negative controls underwent 5–10 additional PCR cycles relative to biological specimens to capture low levels of bacterial template. We utilized both the frequency and prevalence method within the R package decontam (v1.8.0) to identify contaminating ASVs.^48^ Using the “frequency” method, isContaminant(phyloseq_object, method = “frequency”, conc = “qubit”,threshold = 0.05), two ASVs were identified (Supplementary figure 6S). However, these ASVs were present at a very low abundance and only present in two samples. Furthermore, these ASVs were assigned to Clostridiales and Burkholderiales which are known gut taxa and not indicative of contamination (Supplementary table 7). Using the “prevalence” method, isContaminant(phyloseq_object, method = “prevalence”, neg = “is.neg”,threshold = 0.05), we identified seven contaminating ASVs (Supplementary table 8). However, these ASVs were only identified in three of our samples and only contributed between 2–6 reads to the samples. Thus, we treated them negligibly.

## Supplementary Material

Supplemental Material

## References

[1] US Preventive Services Task Force, Bibbins-Domingo K, Grossman DC, Curry SJ, Davidson KW, Epling JW Jr, García FAR, Gillman MW, Harper DM, Kemper AR, *et al*. Screening for colorectal cancer: US preventive services task force recommendation statement. JAMA. 2016;315:2564–2575. doi:10.1001/jama.2016.5989 27304597

[2] Fearon ER, Vogelstein B. A genetic model for colorectal tumorigenesis. Cell. 1990;61:759–10. doi:10.1016/0092-8674(90)90186-I. 2188735

[3] Stryker SJ, Wolff BG, Culp CE, Libbe SD, Ilstrup DM, MacCarty RL. Natural history of untreated colonic polyps. Gastroenterology. 1987;93:1009–1013. doi:10.1016/0016-5085(87)90563-4. 3653628

[4] Flemer B, Lynch DB, Brown JMR, Jeffery IB, Ryan FJ, Claesson MJ, O’Riordain M, Shanahan F, O’Toole PW. Tumour-associated and non-tumour-associated microbiota in colorectal cancer. Gut. 2017;66:633–643. doi:10.1136/gutjnl-2015-309595. 26992426 PMC5529966

[5] Thomas AM, Manghi P, Asnicar F, Pasolli E, Armanini F, Zolfo M, Beghini F, Manara S, Karcher N, Pozzi C, *et al*. Metagenomic analysis of colorectal cancer datasets identifies cross-cohort microbial diagnostic signatures and a link with choline degradation. Nat Med. 2019;25:667–678. doi:10.1038/s41591-019-0405-7 PMC9533319 30936548

[6] Raskov H, Burcharth J, Pommergaard HC. Linking gut microbiota to colorectal cancer. J Cancer. 2017;8:3378–3395. doi:10.7150/jca.20497. 29151921 PMC5687151

[7] Sommer F, Bäckhed F. The gut microbiota–masters of host development and physiology. Nat Rev Microbiol. 2013;11:227–238. doi:10.1038/nrmicro2974. 23435359

[8] Marshall BJ, Warren JR. Unidentified curved bacilli in the stomach of patients with gastritis and peptic ulceration. Lancet. 1984;1:1311–1315. 10.1016/s0140-6736(84)91816-6 6145023

[9] Wang F, Meng W, Wang B, Qiao L. Helicobacter pylori-induced gastric inflammation and gastric cancer. Cancer Lett. 2014;345(196–202). doi:10.1016/j.canlet.2013.08.016. 23981572

[10] Bullman S, Pedamallu CS, Sicinska E, Clancy TE, Zhang X, Cai D, Neuberg D, Huang K, Guevara F, Nelson T, *et al*. Analysis of science. 2017;358:1443–1448. doi:10.1126/science.aal5240. PMC5823247 29170280

[11] Pleguezuelos-Manzano C, Puschhof J, Rosendahl Huber A, Van Hoeck A, Wood HM, Nomburg J, Gurjao C, Manders F, Dalmasso G, Stege PB, *et al*. Mutational signature in colorectal cancer caused by genotoxic pks. Nature. 2020;580:269–273. doi:10.1038/s41586-020-2080-8. 32106218 PMC8142898

[12] Louis P, Hold GL, Flint HJ. The gut microbiota, bacterial metabolites and colorectal cancer. Nat Rev Microbiol. 2014;12:661–672. doi:10.1038/nrmicro3344. 25198138

[13] Peters BA, Dominianni C, Shapiro JA, Church TR, Wu J, Miller G, Yuen E, Freiman H, Lustbader I, Salik J, *et al*. The gut microbiota in conventional and serrated precursors of colorectal cancer. Microbiome. 2016;4(69). doi:10.1186/s40168-016-0218-6 PMC5203720 28038683

[14] Hibberd AA, Lyra A, Ouwehand AC, Rolny P, Lindegren H, Cedgård L, Wettergren Y. Intestinal microbiota is altered in patients with colon cancer and modified by probiotic intervention. BMJ Open Gastroenterol. 2017;4:e000145. doi:10.1136/bmjgast-2017-000145. PMC5609083 28944067

[15] Lu Y, Chen J, Zheng J, Hu G, Wang J, Huang C, Lou L, Wang X, & Zeng Y. *et al*. Mucosal adherent bacterial dysbiosis in patients with colorectal adenomas. Sci Rep. 2016;6: 26337. doi:10.1038/srep26337 PMC4872055 27194068

[16] Weir TL, Manter DK, Sheflin AM, Barnett BA, Heuberger AL, Ryan EP. Stool microbiome and metabolome differences between colorectal cancer patients and healthy adults. PLoS One. 2013;8:e70803. doi:10.1371/journal.pone.0070803. 23940645 PMC3735522

[17] Ahn J, Sinha R, Pei Z, Dominianni C, Wu J, Shi J, Goedert JJ, Hayes RB, Yang L. Human gut microbiome and risk for colorectal cancer. J Natl Cancer Inst. 2013;105:1907–1911. doi:10.1093/jnci/djt300. 24316595 PMC3866154

[18] Nakatsu G, Li X, Zhou H, Sheng J, Wong SH, Wu WKK, Ng SC, Tsoi H, Dong Y, Zhang N, *et al*. Gut mucosal microbiome across stages of colorectal carcinogenesis. Nat Commun. 2015;6:8727. doi:10.1038/ncomms9727 PMC4640069 26515465

[19] Drewes JL, White JR, Dejea CM, Fathi P, Iyadorai T, Vadivelu J, Roslani AC, Wick EC, Mongodin EF, Loke MF, *et al*. High-resolution bacterial 16S rRNA gene profile meta-analysis and biofilm status reveal common colorectal cancer consortia. NPJ Biofilms Microbiomes. 2017;3(34). doi:10.1038/s41522-017-0040-3 PMC5707393 29214046

[20] Flemer B, Warren RD, Barrett MP, Cisek K, Das A, Jeffery IB, Hurley E, O'Riordain M, Shanahan F, O'Toole PW. The oral microbiota in colorectal cancer is distinctive and predictive. Gut. 2017;67(8):1454–1463. doi:10.1136/gutjnl-2017-314814. PMC6204958 28988196

[21] Yang Y, Cai Q, Shu XO, Steinwandel MD, Blot WJ, Zheng W, Long J. Prospective study of oral microbiome and colorectal cancer risk in low-income and African American populations. Int J Cancer. 2019;144(10):2381–2389. doi:10.1002/ijc.31941. PMC6430704 30365870

[22] Punt CJ, Koopman M, Vermeulen L. From tumour heterogeneity to advances in precision treatment of colorectal cancer. Nat Rev Clin Oncol. 2017;14:235–246. doi:10.1038/nrclinonc.2016.171. 27922044

[23] Hewett DG, Kaltenbach T, Sano Y, Tanaka S, Saunders BP, Ponchon T, Soetikno R, Rex DK. Validation of a simple classification system for endoscopic diagnosis of small colorectal polyps using narrow-band imaging. Gastroenterology e591. 2012;143:599–607. doi:10.1053/j.gastro.2012.05.006. 22609383

[24] The Paris endoscopic classification of superficial neoplastic lesions: esophagus, stomach, and colon: November 30 to December 1, 2002. Gastrointest Endosc. 2003;58:S3–43. doi:10.1016/s0016-5107(03)02159-x. 14652541

[25] Kudo S, Tamura S, Nakajima T, Yamano H-O, Kusaka H, Watanabe H. Diagnosis of colorectal tumorous lesions by magnifying endoscopy. Gastrointest Endosc. 1996;44:8–14. doi:10.1016/S0016-5107(96)70222-5. 8836710

[26] Kudo S, Hirota S, Nakajima T, Hosobe S, Kusaka H, Kobayashi T, Himori M, Yagyuu A. Colorectal tumours and pit pattern. J Clin Pathol. 1994;47:880–885. doi:10.1136/jcp.47.10.880. 7962600 PMC502170

[27] Hamilton SR, Aaltonen LA. *World Health Organization classification of tumours. Pathology and genetics of tumours of the digestive system*. Lyon: IARC Press; 2000. p. 108.

[28] Konda K, Konishi K, Yamochi T, Ito YM, Nozawa H, Tojo M, Shinmura K, Kogo M, Katagiri A, Kubota Y, *et al*. Distinct molecular features of different macroscopic subtypes of colorectal neoplasms. PLoS One. 2014;9:e103822. doi:10.1371/journal.pone.0103822. 25093594 PMC4122357

[29] Jones HG, Jenkins G, Williams N, Griffiths P, Chambers P, Beynon J, Harris D. Genetic and epigenetic intra-tumour heterogeneity in colorectal cancer. World J Surg. 2017;41:1375–1383. doi:10.1007/s00268-016-3860-z. 28097409 PMC5394146

[30] Wu Y, Shi L, Li Q, Wu J, Peng W, Li H, Chen K, Ren Y, Fu X. Microbiota diversity in human colorectal cancer tissues is associated with clinicopathological features. Nutr Cancer. 2019;71:214–222. doi:10.1080/01635581.2019.1578394. 30843732

[31] Warren RL, Freeman DJ, Pleasance S, Watson P, Moore RA, Cochrane K, Allen-Vercoe E, Holt RA. Co-occurrence of anaerobic bacteria in colorectal carcinomas. Microbiome. 2013;1:16. doi:10.1186/2049-2618-1-16. 24450771 PMC3971631

[32] Harada T, Yamamoto E, Yamano H-O, Aoki H, Matsushita H-O, Yoshikawa K, Takagi R, Harada E, Tanaka Y, Yoshida Y, *et al*. Surface microstructures are associated with mutational intratumoral heterogeneity in colorectal tumors. J Gastroenterol. 2018;53(12):1241–1252. doi:10.1007/s00535-018-1481-z. 29948303

[33] Love MI, Huber W, Anders S. Moderated estimation of fold change and dispersion for RNA-seq data with DESeq2. Genome Biol. 2014;15(550). doi:10.1186/s13059-014-0550-8. PMC4302049 25516281

[34] Weiss S, Xu ZZ, Peddada S, Amir A, Bittinger K, Gonzalez A, Lozupone C, Zaneveld JR, Vázquez-Baeza Y, Birmingham A, *et al*. Normalization and microbial differential abundance strategies depend upon data characteristics. Microbiome. 2017;5(27). doi:10.1186/s40168-017-0237-y PMC5335496 28253908

[35] Schmidt TS, Hayward MR, Coelho LP, Li SS, Costea PI, Voigt AY, Wirbel J, Maistrenko OM, Alves RJ, Bergsten E, *et al*. Extensive transmission of microbes along the gastrointestinal tract. Elife. 2019;8. doi:10.7554/eLife.42693. PMC6424576 30747106

[36] Modi SR, Collins JJ, Relman DA. Antibiotics and the gut microbiota. J Clin Invest. 2014;124:4212–4218. doi:10.1172/JCI72333. 25271726 PMC4191029

[37] Ghosh TS, Rampelli S, Jeffery IB, Santoro A, Neto M, Capri M, Giampieri E, Jennings A, Candela M, Turroni S, *et al*. Mediterranean diet intervention alters the gut microbiome in older people reducing frailty and improving health status: the NU-AGE 1-year dietary intervention across five European countries. Gut. 2020;69(7):1218–1228. doi:10.1136/gutjnl-2019-319654. 32066625 PMC7306987

[38] Tomkovich S, Dejea CM, Winglee K, Drewes JL, Chung L, Housseau F, Pope JL, Gauthier J, Sun X, Mühlbauer M, *et al*. Human colon mucosal biofilms from healthy or colon cancer hosts are carcinogenic. J Clin Invest. 2019;130:1699–1712. doi:10.1172/JCI124196 PMC6436866 30855275

[39] Ternes D, Karta J, Tsenkova M, Wilmes P, Haan S, Letellier E. Microbiome in colorectal cancer: how to get from meta-omics to mechanism? Trends Microbiol. 2020;28:401–423. doi:10.1016/j.tim.2020.01.001. 32298617

[40] Abed J, Emgård JM, Zamir G, Faroja M, Almogy G, Grenov A, Sol A, Naor R, Pikarsky E, Atlan K, *et al*. Fap2 mediates Fusobacterium nucleatum colorectal adenocarcinoma enrichment by binding to tumor-expressed Gal-GalNAc. Cell Host Microbe. 2016;20:215–225. doi:10.1016/j.chom.2016.07.006. 27512904 PMC5465824

[41] Brennan CA, Garrett WS. Fusobacterium nucleatum – symbiont, opportunist and oncobacterium. Nat Rev Microbiol. 2019;17(156–166):156–166. doi:10.1038/s41579-018-0129-6. 30546113 PMC6589823

[42] Di Filippo S, Delahaye F, Semiond B, Celard M, Henaine R, Ninet J, Sassolas F, Bozio A. Current patterns of infective endocarditis in congenital heart disease. Heart. 2006;92:1490–1495. doi:10.1136/hrt.2005.085332. 16818488 PMC1861050

[43] Jalanka J, Salonen A, Salojärvi J, Ritari J, Immonen O, Marciani L, Gowland P, Hoad C, Garsed K, Lam C, *et al*. Effects of bowel cleansing on the intestinal microbiota. Gut. 2015;64:1562–1568. doi:10.1136/gutjnl-2014-307240. 25527456

[44] Murphy CL, O’Toole PW, Shanahan F. The gut microbiota in causation, detection, and treatment of cancer. Am J Gastroenterol. 2019;114(7):1036–1042. doi:10.14309/ajg.0000000000000075. 30848738

[45] Veziant J, Poirot K, Chevarin C, Cassagnes L, Sauvanet P, Chassaing B, Robin F, Godfraind C, Barnich N, Pezet D, *et al*. Prognostic value of a combination of innovative factors (gut microbiota, sarcopenia, obesity, metabolic syndrome) to predict surgical/oncologic outcomes following surgery for sporadic colorectal cancer: a prospective cohort study protocol (METABIOTE). BMJ Open. 2020;10:e031472. doi:10.1136/bmjopen-2019-031472. PMC6955509 31915159

[46] Kuczynski J, Stombaugh J, Walters WA, González A, Caporaso JG, Knight R. Using QIIME to analyze 16S rRNA gene sequences from microbial communities. Curr Protoc Microbiol. 2012;27(1). **Chapter 1**, Unit 1E.5.. 10.1002/9780471729259.mc01e05s27. PMC4477843 23184592

[47] Caporaso JG, Kuczynski J, Stombaugh J, Bittinger K, Bushman FD, Costello EK, Fierer N, Peña AG, Goodrich JK, Gordon JI, *et al*. QIIME allows analysis of high-throughput community sequencing data. Nat Methods. 2010;7:335–336. doi:10.1038/nmeth.f.303. 20383131 PMC3156573

[48] Davis NM, Proctor DM, Holmes SP, Relman DA, Callahan BJ. Simple statistical identification and removal of contaminant sequences in marker-gene and metagenomics data. Microbiome. 2018;6(226). doi:10.1186/s40168-018-0605-2. PMC6298009 30558668

