# Supplementary material for: Mapping the colorectal tumor microbiota
Source: Gut Microbes. 2021 May 25;13(1):1920657. doi: 10.1080/19490976.2021.1920657 (PMC8158024; doi:10.1080/19490976.2021.1920657)
Supplement: Supplemental Material [file KGMI_A_1920657_SM7541.zip › Document.rtf]

Supplementary figure 1. Variance explained by first 10 PCoA axes.  Bar plot displaying level of variance of variance explained by each access with regard to unweighted Unifrac distance

Supplementary figure 2. Bar plot of the difference in Beta-diversity distance between the microbiota of indicated biopsy sites and paired buccal swab microbiota from the same subject. (A) Unifrac distance (B) Bray–Curtis (C) Jaccard. Kruskal–Wallis test was used to calculate p-values

Supplementary figure 3. Rarefaction Curve. Number of reads on x-axis. Number of unique ASV sequences. Blue lines indicate saliva samples. Red lines indicates colonic biopsy samples.

Supplementary figure 4. Bar plot displaying the difference between Inter-individuals versus Intra-individual variation in alpha-diversity (A) Observed species (B) Phylogenetic diversity (C) Simpson's Diversity Index (D) Shannon index

Supplementary figure 5. Taxonomic bar plot of the proportional relative abundance of genera within controls samples. KC-30 denotes AllPrep DNA kit mock extraction followed by 30 cycle 16s gene PCR amplification. KC-35 denotes AllPrep DNA kit mock extraction followed by 35 cycle 16s gene PCR amplification. pcr-30 denotes mock amplfcation (just water) of the 16s gene. ps-n-30 denotes DNeasy PowerSoil Kit mock extraction followed by 30 cycle 16s gene PCR amplification. 


Supplementary figure 6. Decontam frequency graph. X axis equals concentration of sample before normalization. Y-axis equals frequency of ASV. Each dot represents a sample. 
