# Supplementary material for: Mapping the colorectal tumor microbiota
Source: Gut Microbes. 2021 May 25;13(1):1920657. doi: 10.1080/19490976.2021.1920657 (PMC8158024; doi:10.1080/19490976.2021.1920657)
Supplement: Supplemental Material [file KGMI_A_1920657_SM7541.zip › Supplementary data.docx]

| PCoA axis | P-value |
| --- | --- |
| 1 | 0.0000017603 |
| 2 | 0.0000023873 |
| 3 | 0.0000266130 |
| 4 | 0.0000196660 |
| 5 | 0.1453059839 |
| 6 | 0.2208569369 |
| 7 | 0.9189331198 |
| 8 | 0.8767527693 |
| 9 | 0.7447672631 |
| 10 | 0.9402627020 |

Supplementary table 1. Association between PCoA axes and Patient ID. P value calculated using Kruskal–Wallis test

| PCoA axis | P-value |
| --- | --- |
| 1 | 0.9997487 |
| 2 | 0.9977087 |
| 3 | 0.9787245 |
| 4 | 0.9781814 |
| 5 | 0.2440231 |
| 6 | 0.1786758 |
| 7 | 0.5677194 |
| 8 | 0.4210597 |
| 9 | 0.3520217 |
| 10 | 0.1317221 |

Supplementary table 2. Association between PCoA axes and sample site. P value calculated using Kruskal–Wallis test

| Beta-diversity metric | P-value | R squared |
| --- | --- | --- |
| Weighted unifrac | 0.001 | 0.868 |
| Unweighted unifrac | 0.001 | 0.715 |
| Bray Curtis dissimilarity | 0.001 | 0.852 |
| Jaccard similarity | 0.001 | 0.721 |

Supplementary table 3. Association between beta diversity metrics and Patient ID. P-value and R squared calculated using PERMANOVA.

| Beta-diversity metric | P-value | R squared |
| --- | --- | --- |
| Weighted unifrac | 1 | 0.033 |
| Unweighted unifrac | 1 | 0.047 |
| Bray Curtis dissimilarity | 1 | 0.032 |
| Jaccard similarity | 1 | 0.058 |

Supplementary table 4. Association between beta diversity metrics and Patient ID. P-value and R squared calculated using PERMANOVA.

| Beta-diversity metric | P-value | R squared |
| --- | --- | --- |
| Bray Curtis dissimilarity | 1 | 0.03151 |
| Jaccard similarity | 1 | 0.05768 |

Supplementary table 5. Association between beta diversity metrics and Patient ID with rank-sum normalization. P-value and R squared calculated using PERMANOVA.

| ASV | Order | Genus | Species |
| --- | --- | --- | --- |
| Seq 908 | Burkholderiales | Sutterella | Sutterella stercoricanis |
| Seq 1091 | Clostridiales | unclassified | unclassified |

Supplementary table 7. ASV identified as contamination using the “frequency” method within decontam.

| ASV | Genus | Species |
| --- | --- | --- |
| Seq_4 | Ralstonia | Ralstonia insidiosa |
| Seq_6 | Pseudomonas | unclassified |
| Seq_8 | Pseudomonas | unclassified |
| Seq_30 | Achromobacter | unclassified |
| Seq_243 | Tepidimonas | unclassified |
| Seq_311 | Pseudomonas | unclassified |
| Seq_626 | Propionibacterium | Propionibacterium acnes |

Supplementary table 8. ASV identified as contamination using the “prevalence” method within decontam.
