# Supplementary figures and images for: Mapping the colorectal tumor microbiota
Source: Gut Microbes. 2021 May 25;13(1):1920657. doi: 10.1080/19490976.2021.1920657 (PMC8158024; doi:10.1080/19490976.2021.1920657)

## Variance explained by top ten PCoA axes

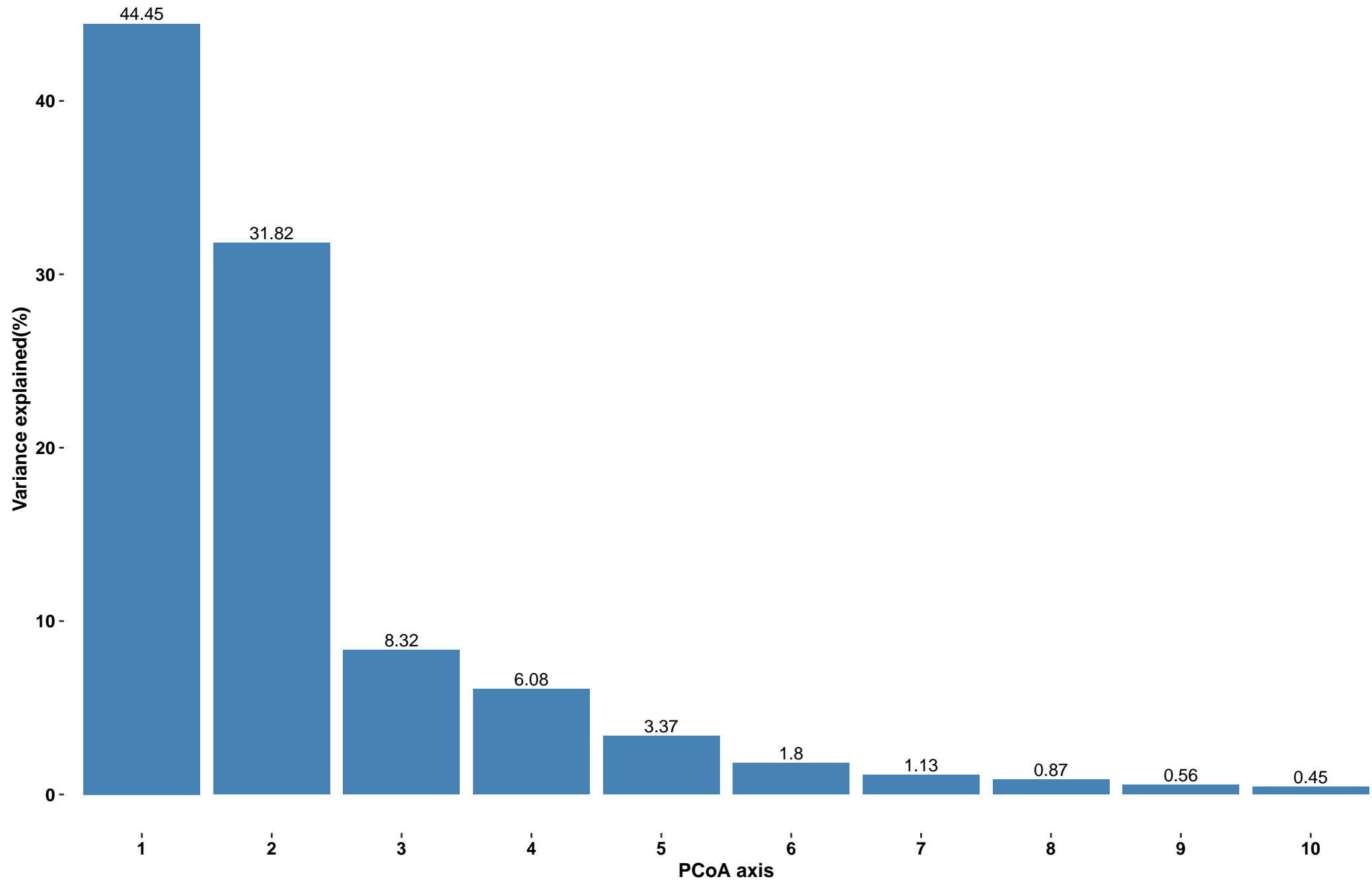

Supplement: Supplemental Material [file KGMI_A_1920657_SM7541.zip › Supplementary Figure 1.pdf]

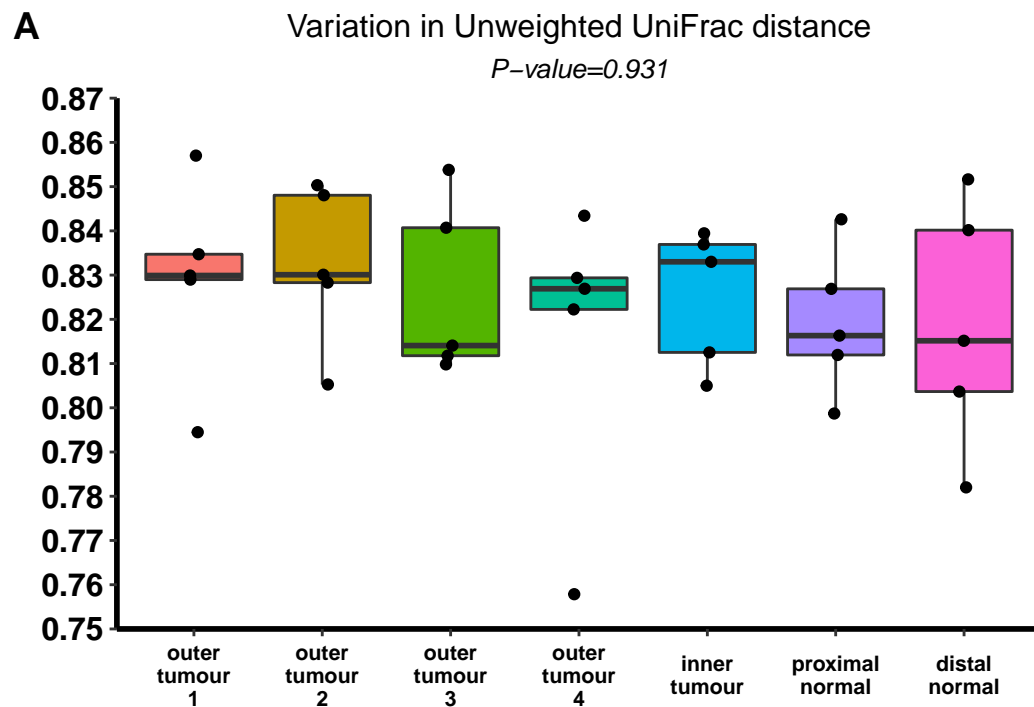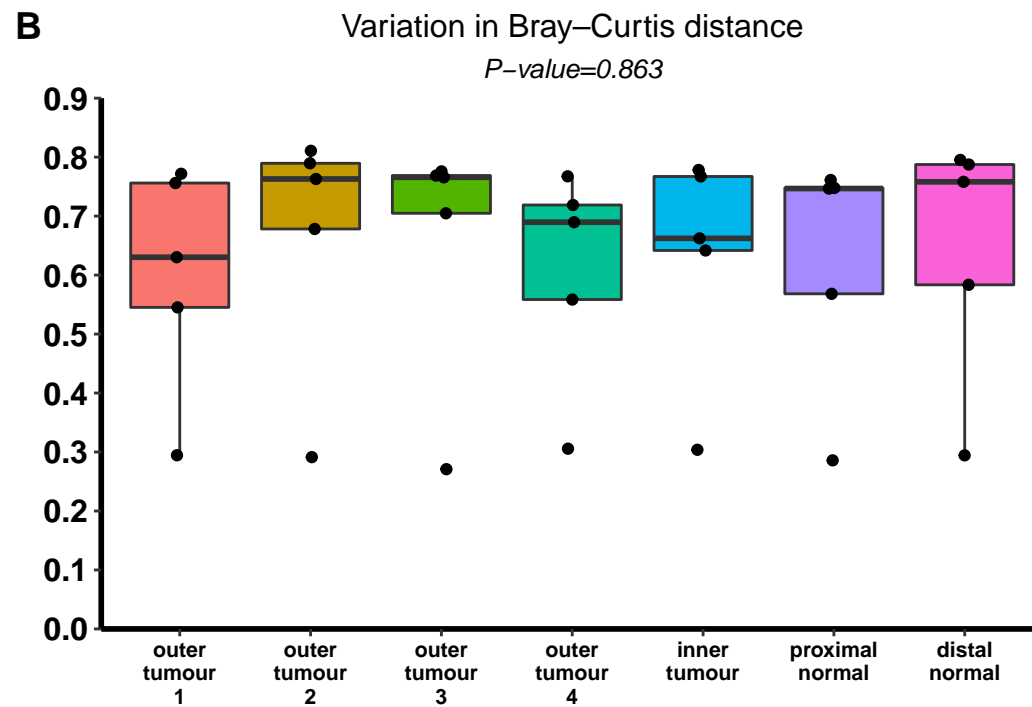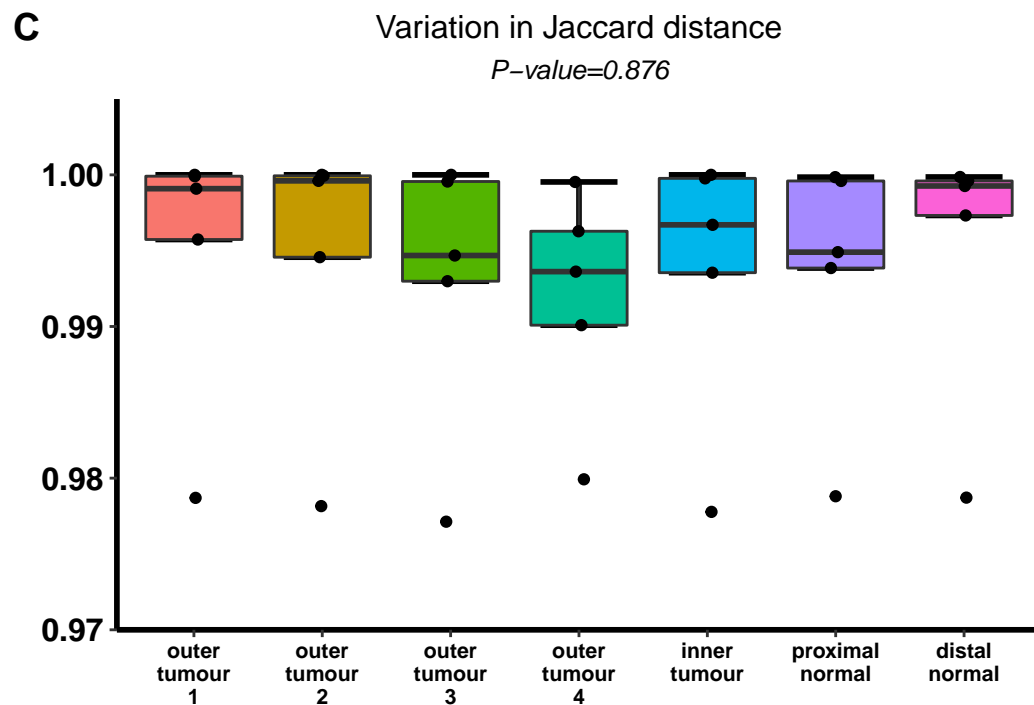

Supplement: Supplemental Material [file KGMI_A_1920657_SM7541.zip › Supplementary Figure 2.pdf]

# Rarefaction Curve

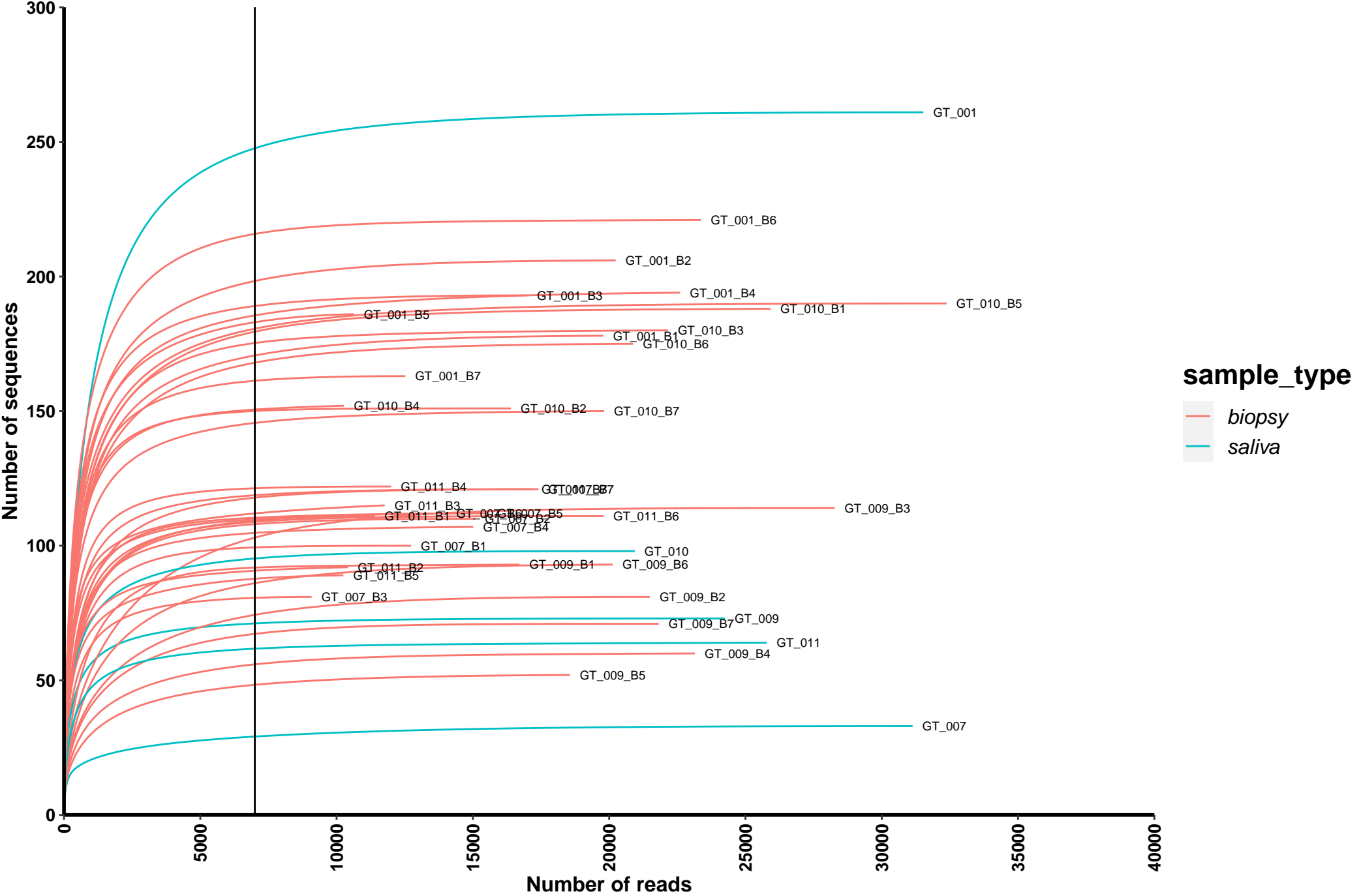

Supplement: Supplemental Material [file KGMI_A_1920657_SM7541.zip › Supplementary Figure 3.pdf]

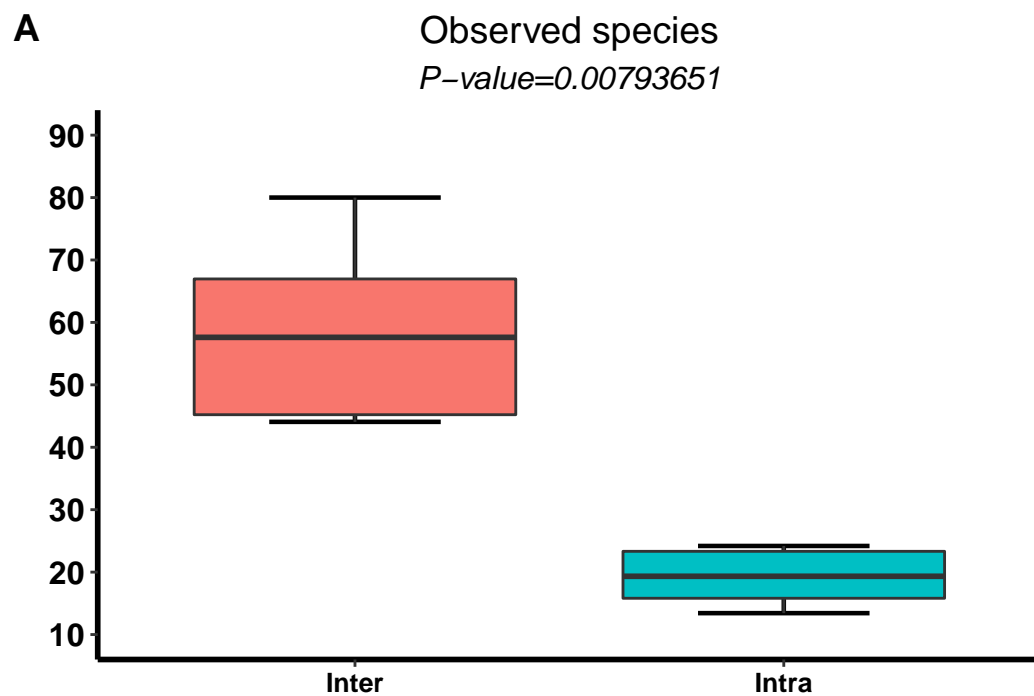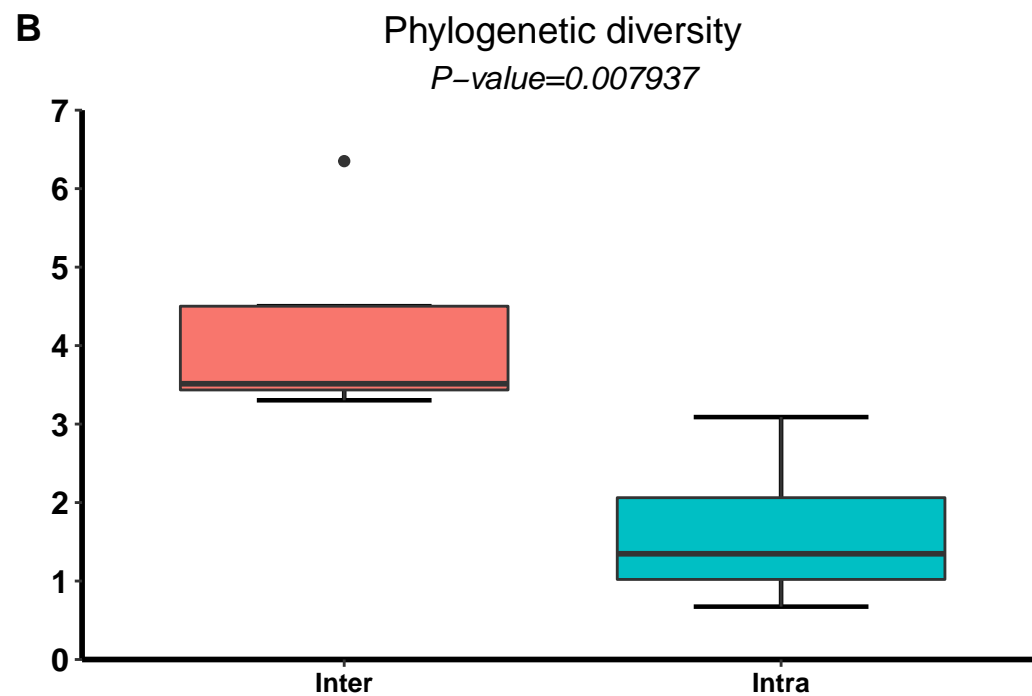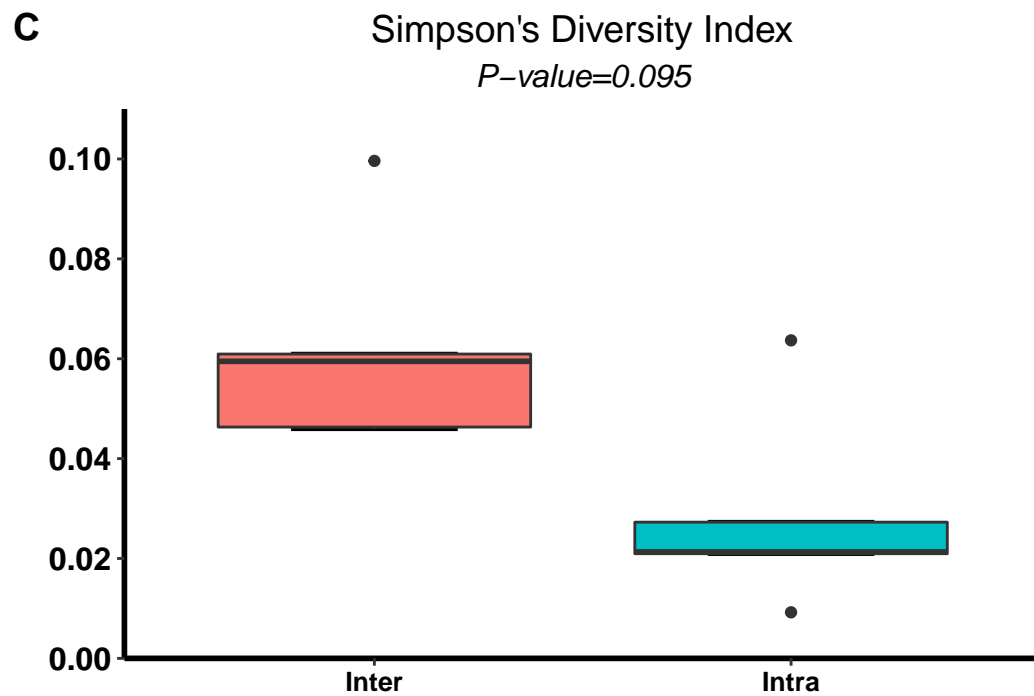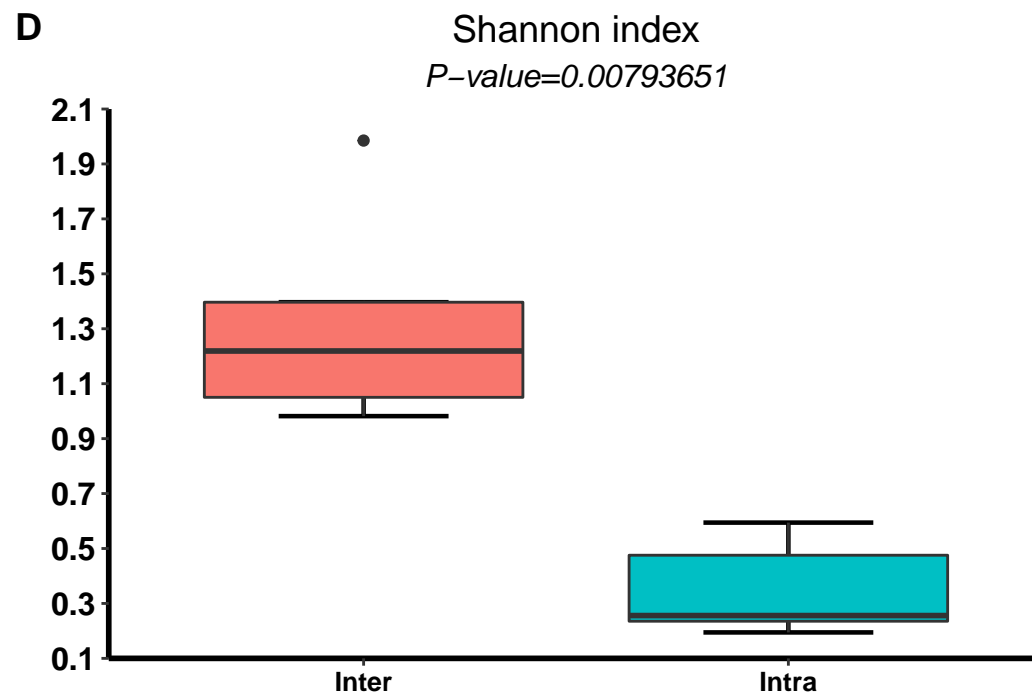

Supplement: Supplemental Material [file KGMI_A_1920657_SM7541.zip › Supplementary Figure 4.pdf]

Controls – genus representation

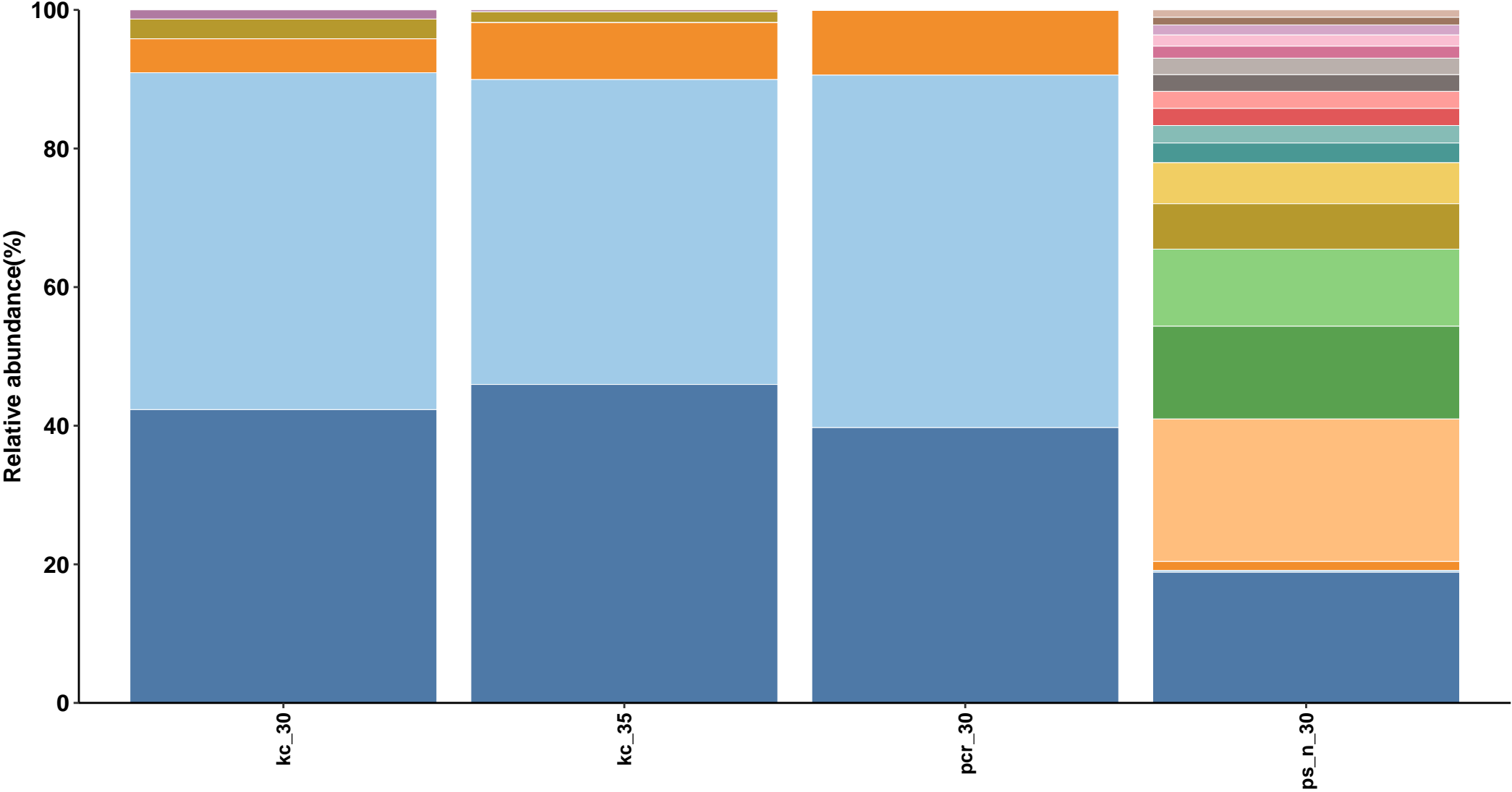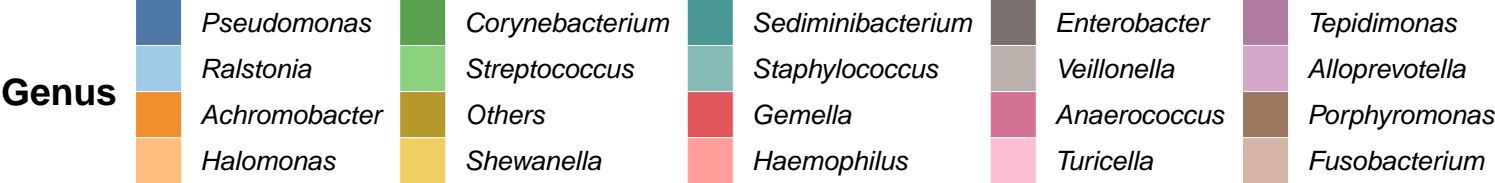

Supplement: Supplemental Material [file KGMI_A_1920657_SM7541.zip › Supplementary Figure 5.pdf]

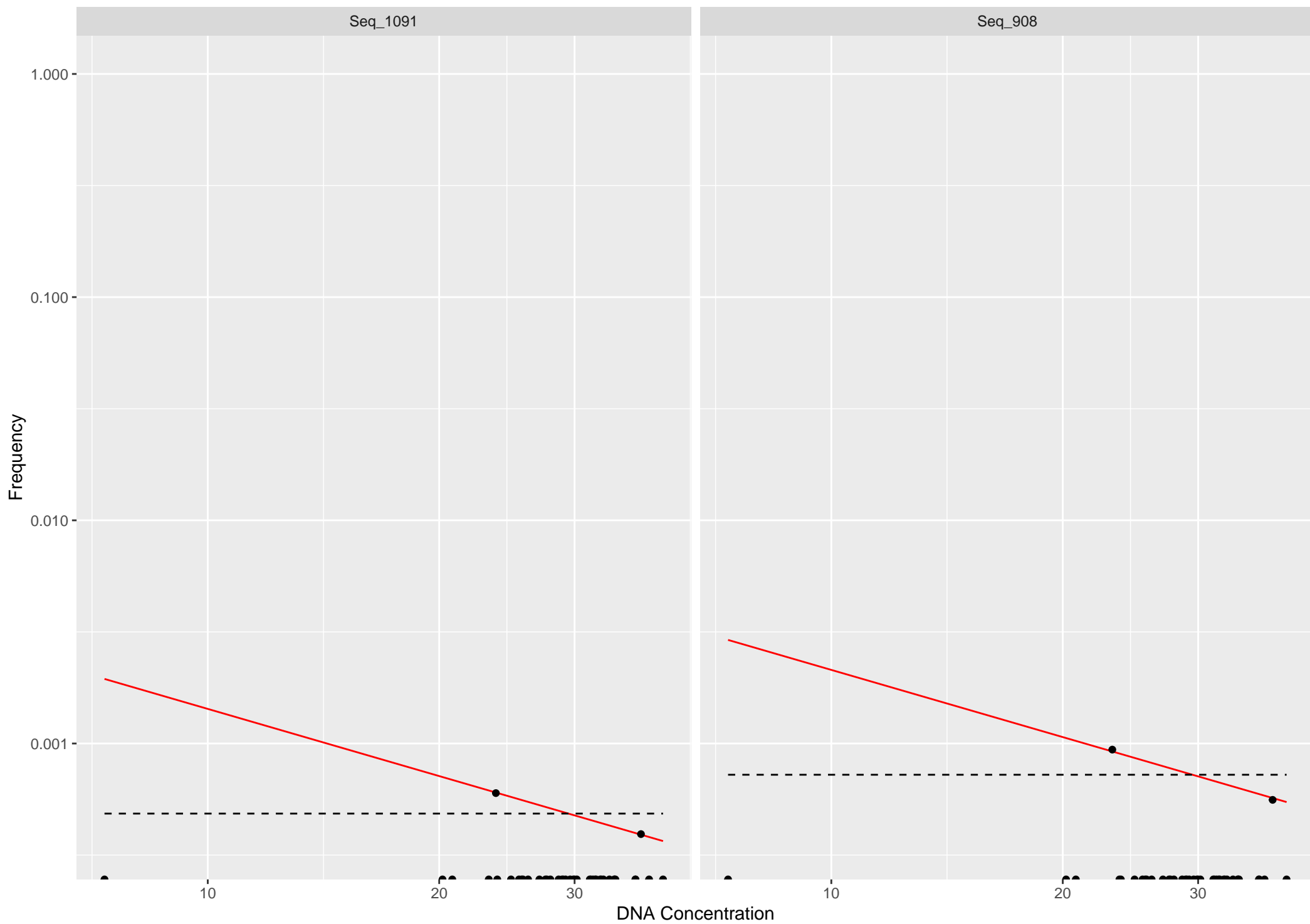

Supplement: Supplemental Material [file KGMI_A_1920657_SM7541.zip › Supplementary Figure 6.pdf]
